# Steroid Responsive Mononeuritis Multiplex in the Cronkhite–Canada Syndrome

**DOI:** 10.3389/fneur.2016.00207

**Published:** 2016-11-17

**Authors:** Y. L. Lo, K. H. Lim, X. M. Cheng, S. Mesenas

**Affiliations:** ^1^Department of Neurology, National Neuroscience Institute, Singapore General Hospital, Singapore; ^2^Due-NUS Medical School, Singapore; ^3^Department of Anatomical Pathology, Singapore General Hospital, Singapore; ^4^Department of Gastroenterology and Hepatology, Singapore General Hospital, Singapore

**Keywords:** Cronkhite–Canada syndrome, neuropathy, mononeuritis multiplex, steroids, autoimmune

## Abstract

The Cronkhite–Canada syndrome (CCS) is a rare disorder of unknown origin characterized by generalized gastrointestinal polyposis, alopecia, hyperpigmentation, and onychodystrophy. We report a case of CCS with concomitant presentation of mononeuritis multiplex. The electrophysiological findings and steroid responsiveness suggests presence of an autoimmune mechanism.

The Cronkhite–Canada syndrome (CCS) is a rare disorder of unknown origin characterized by generalized gastrointestinal polyposis, alopecia, hyperpigmentation, and onychodystrophy (1). Patients develop protein-losing enteropathy and progressive malnutrition. Neurological complications in CCS are seldom reported. To our knowledge, only one case report of peripheral neuropathy in association with CCS has been published (2). Here, we report an unusual case of CCS with concomitant presentation of mononeuritis multiplex (MM). Informed and written consent has been obtained from the patient for the purpose of this report.

## Case Report

A 71-year-old man with previous medical histories of hypertension, hemorrhoids, and glaucoma first presented with weight loss, hair loss, abdominal colic, altered taste, and diarrhea for a 4-month duration. He denied having melena, hemetemesis, or blood in his stools.

He also complained of having hypoesthesia and paresthesia in his hands and feet simultaneously 6 months later. However, no pain, weakness, rashes, joint swelling, and fever were noted. His vision, speech, hearing, and smell sensation were unchanged.

Clinical examination revealed he was not jaundiced, pale, or cachectic. His abdomen was soft and not distended. There was no palpable hepatosplenomegaly or lymphadenopathy.

Neurological examination revealed mild motor weakness (4/5 on the MRC scale) of the finger abduction, ankle dorsiflexion, and plantar flexion, involving the left side more severely. However, deep tendon reflexes were diminished in both ankles and sensory testing revealed diminished touch and temperature sensation of both hands and feet. Vibration and position sense were intact. Gait and cerebellar system testing were normal.

Hematological investigations performed initially showed normal blood counts and electrolytes, but hypoalbuminemia (5 μmol/L) without transaminitis. Thyroid function, vitamin B12, and folate levels were unremarkable. Autoimmune tests, including antinuclear, anti-Smith, anti-smooth muscle, Ro, La, ribonuclear protein, anti-double stranded DNA, and anti-Jo1 titers were not elevated. Hepatitis B and C serology were negative.

CT abdominal scan revealed few diverticula in the colon. CT colonoscopy, however, found extensive nodular appearance of the colonic mucosa and few polyps.

The patient underwent endoscopic examination, whereby gastroscopy showed infiltrative lesions in the stomach and duodenum, while colonoscopy detected multiple strawberry-like hamartomatous polyps in the entire colon and occasional tubulovillous adenomas with low-grade dysplasia. Biopsies of the stomach, duodenum, and colon revealed regenerative and hyperplastic changes with prominent lamina propia odema and increased eosinophils.

In view of his neurological complaints, he underwent a nerve conduction study (NCS) which showed asymmetrically reduced left peroneal sensory, right sural sensory, left peroneal motor, and left tibial motor amplitudes (Tables 1 and 2). In addition, there was significant reduction of bilateral ulnar sensory and motor amplitudes. Sympathetic skin responses were normal in all four limbs.

**Table 1:** **Sensory NCS**.

NCS 1				NCS2		
Nerve	Latency (ms)	Amplitude (μV)	Conduction velocity (m/s)	Latency (ms)	Amplitude (μV)	Conduction velocity (m/s)
L median sensory	2.85 (<3.9)	10.8 (>10)	45.6 (>45)	2.69	31.9	48.3
R median sensory	3.09	10.1	42.1	3.09	24.2	45.3
L ulnar sensory	2.38 (<3)	**4.5** (>7)	46.2 (>45)	2.23	15.6	49.3
R ulnar sensory	2.30	**3.3**	47.8	2.34	15.7	51.3
L peroneal sensory	3.00 (<3.1)	**0.89** (>4)	40.0 (>38)	2.55	6.4	45.1
R peroneal sensory	3.00	5.3	40.0	2.75	7.3	43.6
L sural sensory	2.92 (<3.8)	2.5 (>2.0)	41.1 (>38)	2.71	5.7	44.3
R sural sensory	3.00	**1.9**	40.0	2.22	5.2	45.0

*NCS, nerve conduction study; L, left; R, right; 1, first NCS; 2, second NCS (performed 2 years later)*.

*Normal values indicated in brackets*.

*Abnormal values indicated in bold*.

**Table 2:** **Motor NCS**.

NCS 1				NCS2		
Nerve	Latency (ms)	Amplitude (mV)	Conduction velocity (m/s)	Latency (ms)	Amplitude (mV)	Conduction velocity (m/s)
L median motor	3.98 (<4.5)	5.9 (>5)	53.2 (>45)	3.28	7.7	51.0
R median motor	4.21	5.2	54.1	4.13	6.6	53.2
L ulnar motor	2.66 (<3.1)	**3.4** (>6)	51.7(>45)	2.83	8.5	52.2
R ulnar motor	2.65	**3.3**	53.9	2.90	9.1	49.7
L tibial motor	4.37 (<5.5)	**1.15** (>2)	38.3 (>38)	3.91	5.0	45.9
R tibial motor	5.21	2.1	41.3	4.04	10.1	44.7
L peroneal motor	4.43 (<4.8)	**1.65** (>2)	35.6 (>38)	3.87	3.8	41.1
R peroneal motor	2.18	2.4	37.3	3.51	3.8	41.8

*NCS, nerve conduction study; L, left; R: right; 1, first NCS; 2, second NCS (performed 2 years later)*.

*Normal values indicated in brackets*.

*Abnormal values indicated in bold*.

He was diagnosed with CCS based on the clinical picture and characteristic histological findings. He was given parenteral nutrition on initial diagnosis. Intravenous hydrocortisone of 100 mg was administered four times a day, and maintenance oral prednisolone 50 mg daily was continued when neuropathy was diagnosed on NCS.

**Figure 1:**
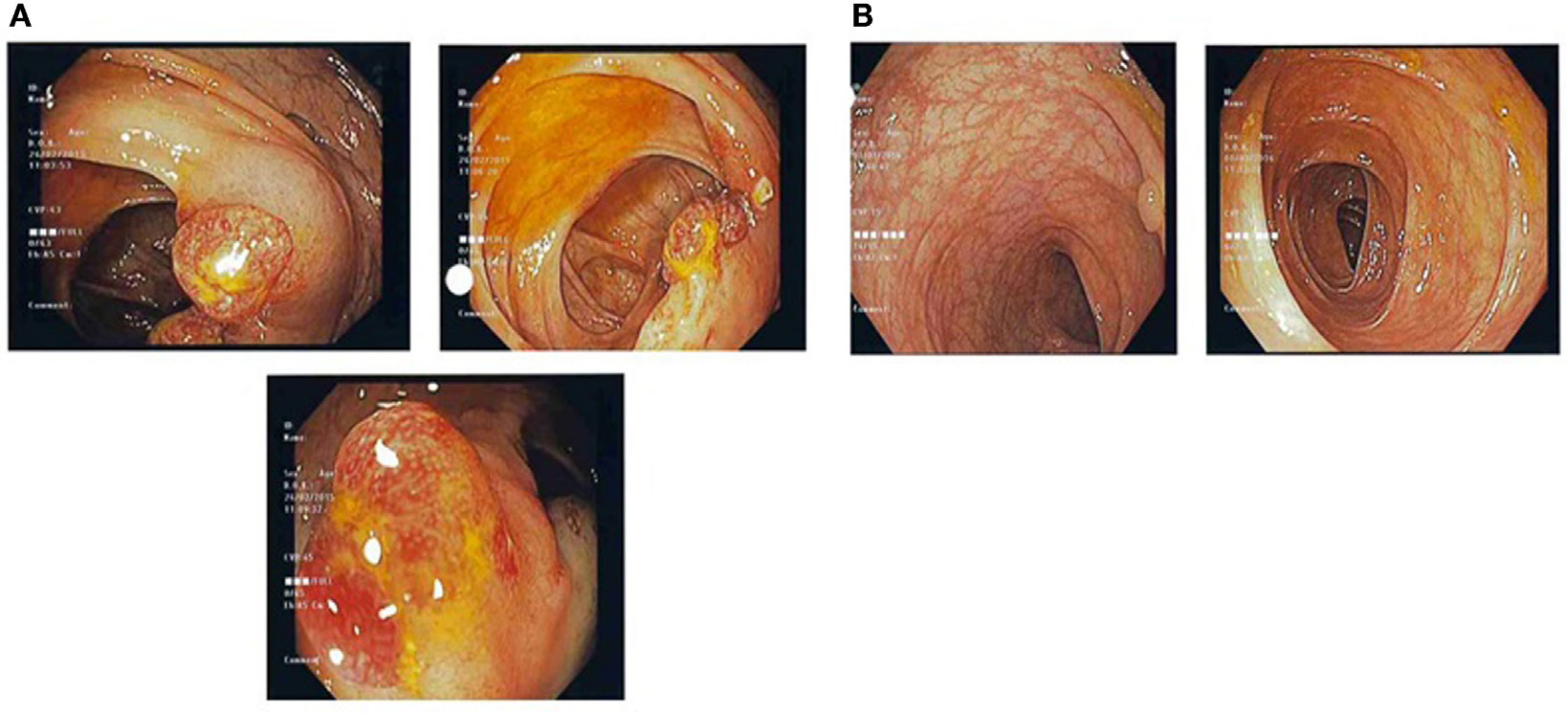
**(A)** Pre-treatment colonic images showing multiple strawberry-like hamartomatous colonic polyps. **(B)** Post-treatment colonic images showing a single tubulovillous adenoma with low-grade dysplasia and resolution of previous hamartomatous polyps.

**Figure 2:**
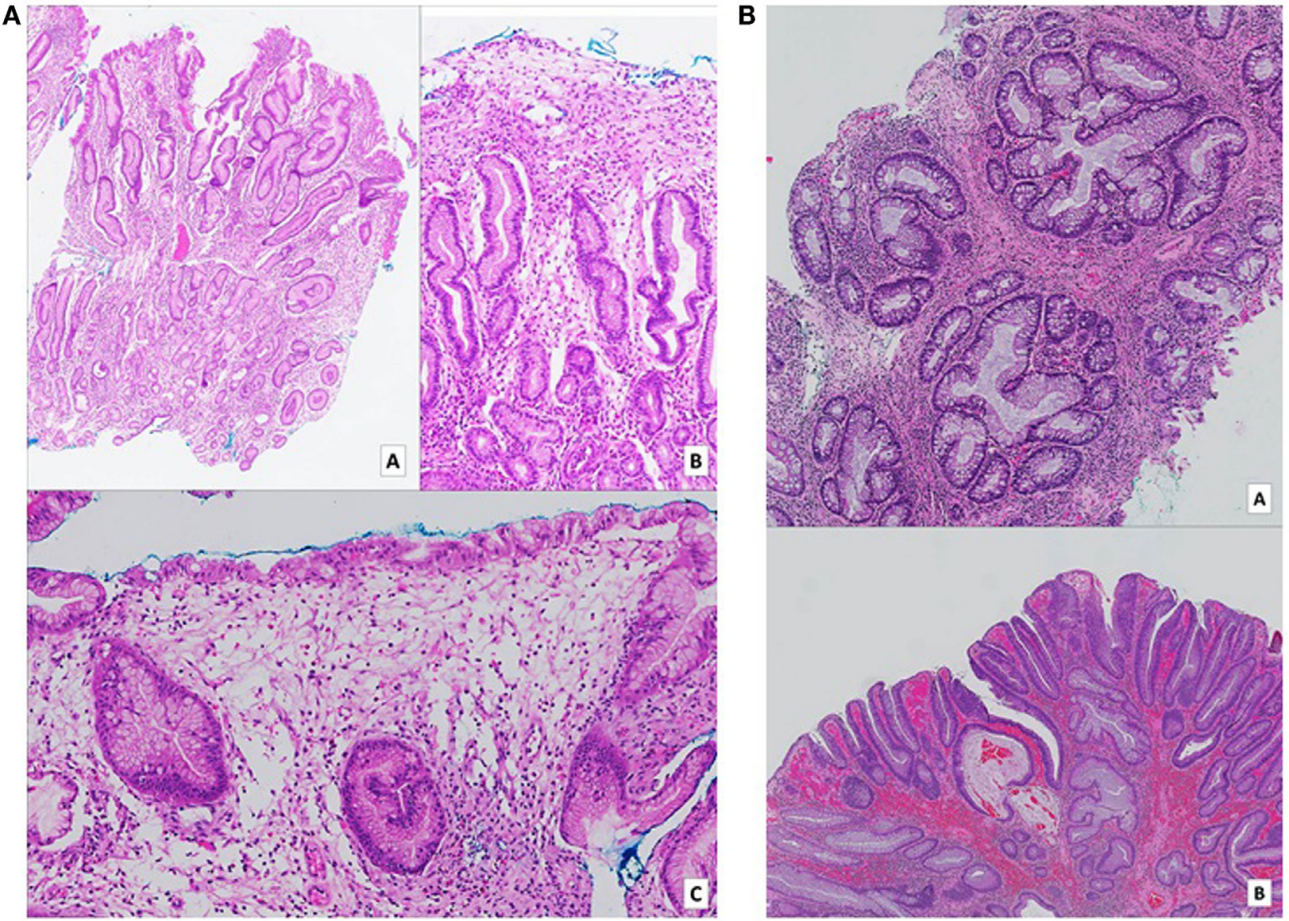
**(A)** Gastric biopsies taken at time of diagnosis. Numerous pieces from various parts of the stomach show similar features. On low (a – H&E, 2× magnification) and medium (b – H&E, 10× magnification) power views, tortuous hyperplastic glands with occasional mild branching and dilatation are identified. A different field (c – H&E, 10× magnification) demonstrates an area of prominent lamina propria edema with a mixed inflammatory cell infiltrate including eosinophils. **(B)** (a – H&E, 5× magnification) (b – H&E, 2× magnification): biopsies of colonic hamartomatous polyps taken a year after diagnosis. The crypts are tortuous, and some are cystically dilated secondary to inspissated mucin. The intervening lamina propria shows an infiltrate of predominantly mononuclear cells, as well as occasional hypertrophic strands of smooth muscle cells. **(B)** Shows a focus of low-grade dysplasia arising on the background of a hamartomatous polyp.

He experienced gradual improvement in gastrointestinal symptoms, taste, appetite, and body weight over a 2-year period. Oral corticosteroids were reduced gradually and azathioprine was added as a steroid-sparing agent. A repeat gastroscopy and colonoscopy revealed normalization of mucosal changes (Figures 1 and 2). The patient also reported reduction of sensory complaints and strength improvement in his limbs. Examination showed residual reduced ankle tendon reflexes and touch sensation in both soles.

A repeat NCS (Tables 1 and 2), 2 years from the initial, demonstrated significant improvement in the above mentioned sensory and motor nerve amplitudes of above 100%.

## Discussion

This report describes a CCS patient presenting with clinical and electrophysiological features of MM, which responded to steroid therapy in tandem with improvement of his gastrointestinal dysfunction. This simultaneous observation suggests that the neurological and gastrointestinal processes share a common pathophysiological process. The extensive investigations performed render nutritional and metabolic factors unlikely causes for his neuropathy. As his neurological symptoms appeared way after parental nutrition was administered, vitamin deficiency as a cause of neuropathy, hence, was unlikely. The impressive response to steroid therapy also supports the hypothesis that CCS is an immune-mediated disease.

Mononeuritis multiplex denotes neuropathy of individual nerves not in a length-dependent or radicular pattern. Clinically, MM can present as asymmetrical, asynchronous sensory, and/or motor polyneuropathy involving two or more separate nerve in random patterns of distribution: bilaterally, distally, proximally, or combinations of these. Hence, it can also be termed as “multiple mononeuropathy,” and autoimmune, paraneneoplastic, and diabetic etiologies are often implicated (3, 4). Electrophysiologically, NCS often shows an axonal neuropathy whereby side-to-side comparison show grossly asymmetric amplitudes (5), as in this patient. A previous study of non-systemic vasculitis reported that most patients exhibited a multifocal or asymmetric, distally accentuated pattern of involvement, particularly affecting the peroneal and ulnar nerves. NCS also showed predominantly axonal, asymmetric, sensorimotor polyneuropathy (6). Other investigators also reported similar findings (7, 8), and these are corroborated by the NCS of our patient. Normal sympathetic skin responses suggest that small fiber-mediated sudomotor sympathetic function is not affected in this CCS patient. In all, the electrophysiological features point to an underlying autoimmune process.

While the exact incidence of etiologies in a cohort of MM patients has not be studied, MM is the most common pattern of involvement in vasculitic neuropathy, to the extent that both terms are often used interchangeably. Indeed, for primary systemic vasculitic neuropathy, up to 85% of cases with polyarteritis nodosa, 80% of cases with Churg-Strauss syndrome, and 40% of cases with Wegener’s granulomatosis have vasculitic neuropathy. However, in several other connective tissue diseases, such as mixed connective tissue disease and sarcoidosis, vasculitic neuropathy occur at a much lower frequency (9).

The cause of CCS is unknown to date. Previously reported associations include systemic lupus erythematosus, scleroderma, hypothyroidism, vitiligo, antinuclear antibodies, and mast cell dysfunction (10), suggesting an autoimmune mechanism at play.

The option of a nerve biopsy was discussed with our patient. Overall, its yield is approximately 60% (8), and sampling error cannot be ruled out in view patchy involvement in MM. Henceforth, he declined initial biopsy and opted for immunotherapy, resulting in significant improvement. A previous case report (2) of a CCS with clinical and NCS features of CCS who underwent sural nerve biopsy did not reveal any specific features apart from reduced myelinated fibers. However, this patient did not receive steroid therapy but was treated with a high protein and caloric diet. The patient demised 5 months after diagnosis.

To date, various treatment of CCS reported include dietary intervention, antibiotics, and immunosuppression, including steroids (11–13). However, the rarity of this condition renders large-scale studies unrealistic. Our patient’s NCS findings of MM point to large fiber involvement with increased likelihood of an underlying autoimmune etiology whereby steroid therapy can be a reasonable option. In addition, CCS has been associated with vitiligo, lupus erythematosus, scleroderma, and hypothyroidism (10), all pointing to an immune-mediated process.

This is the first reported case of steroid responsive MM in the CCS and significant recovery with steroid therapy suggests an autoimmune process underlying this condition.

## Author Contributions

YL: manuscript preparation and clinical care. KL and XC: pathological inputs and clinical care. SM: manuscript inputs, clinical care.

## Conflict of Interest Statement

The authors acknowledge the conditions and report no conflicts of interest.
